# Cost-effectiveness analysis of robotic surgery in healthcare for older individuals: a systematic review based on randomized controlled trials

**DOI:** 10.3389/fpubh.2025.1614654

**Published:** 2025-08-12

**Authors:** Yunkai Tang, Bing Dou

**Affiliations:** ^1^School of Law and Politic, Zhejiang Sci-Tech University, Hangzhou, China; ^2^School of Management, South-Central Minzu University, Wuhan, China

**Keywords:** robotic surgery, older individuals, cost-effectiveness, randomized controlled trials, health economics, systematic review and meta-analysis

## Abstract

**Objectives:**

The objective of this research is to assess the economic viability of robotic interventions in the management of healthcare for the aging demographic by conducting a Systematic Review and Meta-Analysis (SR/MA) of Randomized Controlled Trials (RCTs).

**Methods:**

We conducted a SR/MA following the PRISMA guidelines and the Cochrane Collaboration recommendations. Studies of interest were pinpointed within various databases, encompassing PubMed, Web of Science, and the Cochrane Library, up until the cutoff date of November 2024. Inclusion criteria were based on the PICOS framework, focusing on older patients (≥60 years old), robotic or robot-assisted interventions, cost-related outcomes, and RCTs. The data were subjected to statistical evaluation via Stata 17 software, wherein mean discrepancies (MD) and standardized mean discrepancies (SMD) were computed, accompanied by 95% confidence intervals (CI) for precision. Sensitivity analyses were conducted to address heterogeneity.

**Results:**

Five RCTs involving 666 patients were included. The findings revealed that robotic surgery incurred higher total costs compared to traditional surgical approaches (MD = 1316.38, 95% CI 10.68–2622.08; *p* = 0.048, *I^2^* = 92.5%). Subgroup analysis revealed that operating room costs were notable higher for robotic surgeries (MD = 1151.14, 95% CI 824.63–1477.64; *p* = 0.000, *I^2^* = 0.0%), while hospitalization costs were lower but not statistically significant. Quality-adjusted life year (QALY) gains were statistically significant for robotic surgeries (MD = 0.01, 95% CI 0.00–0.02; *p* = 0.010, *I^2^* = 0.0%). Incremental cost-effectiveness analyses showed that robotic surgery achieved cost-effectiveness in some cases, with incremental costs per QALY ranging from $14,925.62 to $28,860, both below the commonly accepted threshold of $50,000.

**Conclusion:**

Robotic surgery demonstrate potential cost-effectiveness in older individuals, particularly by improving QALY and reducing long-term healthcare costs. However, the high initial investment remains a significant barrier to adoption. Future research should focus on standardizing economic evaluations, exploring specific applications of robotic therapies, and addressing long-term cost and clinical outcomes to better inform healthcare policy and practice.

## 1 Introduction

The integration of robotic technology into healthcare has, to a certain extent, transformed the treatment and management of various diseases. With the global aging population, the prevalence of chronic illnesses, the rising incidence of cancer, and the increasing demand for efficient and cost-effective healthcare solutions, robotic therapy is gradually emerging as one of the key technologies to address these challenges (1). Robots used in healthcare can be broadly categorized into surgical robots for minimally invasive procedures such as cancer and gynecologic surgeries, rehabilitation robots that assist in motor function recovery, and socially assistive robots aimed at enhancing emotional well-being and cognitive engagement. Robotic therapies encompass a range of applications from surgical assistance to rehabilitation, offering promising avenues to improve patient outcomes while potentially lowering healthcare costs (2). Especially among the people aged 60 years and above, the total number of cancer cases is expected to rise by 45% between 2010 and 2030, largely driven by the increasing population of older individuals. It is projected that, by 2030, individuals aged 65 and older will represent 70% of all cancer cases (3). The development of robotic technology can help provide precise, personalized treatment plans, improving surgical accuracy, shortening recovery times, and reducing complications. Not only does this enhance the quality of life for older patients, but it also alleviates the burden on healthcare systems (4). More importantly, robotic therapy could lead to gains in Quality-Adjusted Life Years (QALY), improving treatment outcomes and reducing side effects, thereby enhancing the patient’s life quality and extending healthy lifespan (5, 6). This makes robotic therapy in the aging population not only a means of increasing healthcare efficiency and reducing costs but also a way to provide better long-term health outcomes, positioning it as a vital tool to meet the healthcare demands of an aging society.

Globally, due to advances in engineering, artificial intelligence, and the growing emphasis on precision medicine, the application of robotic technology in healthcare has been accelerating. Countries such as the United States, Japan, and members of the European Union have been at the forefront of integrating robotic technology into their healthcare infrastructures, supported by substantial research and development investments (7). In China, although the adoption of robotic technology began later, it is currently undergoing rapid development, with applications including robots for orthopedics surgery, robotic laparoscopic systems, interventional robotic surgery devices, as well as other surgical robots (7). Currently, Systematic reviews and meta-analyses (SR/MA) have shown that robotic treatments are effective in reducing postoperative complications and accelerating recovery times for older patients. However, cost remains a contentious issue, with research showing mixed results regarding the overall economic benefits. Higgins et al. suggest that although robotic surgery provides distinct technological advantages, the cost of consumables is considerably higher compared to laparoscopic surgery (8). Researchers emphasize that the increased cost of robotic surgery can only be justified if it can be proven to offer significant advantages in patient-centered clinical outcomes. Morgan et al. suggest that despite the initial high investment in robotic surgery, the overall surgical cost is expected to decrease as technology advances and experience grows (2).

Additionally, there has been limited assessment of robotic treatment for older patients using a SR/MA approach that integrates data from randomized controlled trials (RCTs). This gap highlights the need for systematic reviews that combine clinical and economic perspectives to guide policy and clinical decision-making. At the same time, healthcare policymakers and practitioners lack strong evidence to guide investment and implementation decisions, which may hinder the widespread adoption of beneficial robotic therapies. Therefore, there is an urgent need to study the economic impact of robot-assisted surgical treatments to ensure their clinical benefits and financial sustainability within healthcare systems.

This study aims to perform a cost-effectiveness evaluation of robotic therapies for older patients using a SR/MA approach based on RCTs, to comprehensively evaluate their economic and clinical benefits. This will provide valuable evidence for policymakers in reaching well-informed decisions on healthcare investments and resource allocation, while also offering crucial insights for clinical practitioners considering the application of this technology.

## 2 Methods

We adhered to the Preferred Reporting Items for Systematic Reviews and Meta-Analyses (PRISMA) guidelines and the Cochrane Collaboration’s recommendations in reporting this SR/MA (9).

### 2.1 Sources of data and search strategy

Two investigators (Y.T. and B.D.) independently searched Web of Science, PubMed, Embase, The Cochrane Library, CNKI, VIP, Wan Fang Data and CBM databases from database construction until December 2024. In addition, we conducted a supplementary inquiry into gray literature and relevant references. The following search terms were used: (“aged” OR “older individuals” OR “older adults” OR “older patients” OR “seniors”) AND (paro or robot* or “social interactive robot*” or “seal robot” or “assistive robot*” or “personal assistive robot*” or “social interactive robot*” or “assistive robot*” or “companion robot*” or “social commitment robot*” or “robot* therapy” or “therapeutic robot*” or “robot interaction” or “personal robot*” or “therapeutic seal robot*”) AND (randomized controlled trial OR controlled clinical trial OR clinical trials OR randomly trial OR randomized OR placebo).

### 2.2 Selection of studies

Two reviewers (Y.T. and B.D.) independently conducted the study selection in two stages: initial screening of titles and abstracts, followed by full-text review for potentially eligible articles. Any discrepancies arising during the selection process were resolved through discussion between the two reviewers. Using the Participants, Intervention, Comparison, Outcome, and Study Design (PICOS) framework, we defined the inclusion and exclusion criteria (10). The following criteria for inclusion were applied: First, in accordance with the World Health Organization (WHO) definition, older individuals are defined as those aged 60 years and above (11). Second, the treatment group consisted of robotic therapy or robot-assisted therapy, while the control group received traditional treatment methods. Third, studies were required to clearly discuss cost-related outcome measures, such as surgical costs, cost-effectiveness analysis, and economic evaluations. Fourthly, the research design is required to be an RCT, ensuring inclusivity without limitations on age, gender, race, or sociodemographic background.

The following types of studies were not included:

1. Studies that included individuals under 60 years of age, in addition to those aged 60 and above.
2. Studies that reported only on outcomes without addressing cost-effectiveness.
3. Reviews, protocols, letters, case reports, abstracts, trial protocols, and studies that failed to report data.
4. Duplicate publications of the same study.

### 2.3 Data collection and management

All identified literature was imported into EndNote X9.0 for management. Following the elimination of duplicate entries, 2 researchers (Y.T. and B.D.), both possessing substantial expertise in the domain, independently reviewed and extracted data in accordance with the predefined criteria for inclusion and exclusion. Any discrepancies emerging from this process were resolved via dialog between the two researchers. The data extracted from the studies that met the inclusion criteria included the following details: the name of the first author, publication year, country, patient condition, sample size, treatment intervention, control intervention, gender ratio, age range, and outcome measures for each included study. To facilitate cross-country comparisons, all cost data were converted to 2023 US dollars. For studies reporting costs in local currencies, we applied the average annual exchange rate published by the International Monetary Fund (IMF) for the corresponding year of the study. When necessary, cost figures were adjusted for inflation using the Consumer Price Index (CPI) of the respective country, followed by conversion to USD using the 2023 exchange rate.

### 2.4 Quality evaluation

Two researchers (Y.T. and B.D.) carried out an independent evaluation of the methodological quality of the studies that were incorporated into the review. Any conflicts arising from this assessment were intended to be resolved through discussion between the two researchers. The assessment of potential bias was conducted using the Cochrane Collaboration’s Risk of Bias tool, focusing on the following domains: the generation of random sequences, the concealment of allocation, the blinding of participants and personnel, the blinding of outcome assessors, incomplete outcome data, selective reporting of outcomes, and other sources of bias (12, 13). Based on these seven domains, studies were classified as having a “low risk of bias” (if all seven domains were assessed as “low”), a “high risk of bias” (if one or more domains were assessed as “high”), or an “unclear risk of bias” (if one or more domains were rated as “unclear,” with no domains categorized as “high”) (14). Discrepancies will be resolved through dialog between the 2 authors.

### 2.5 Data integration and statistical evaluation

Analyses of the statistical data were conducted utilizing Stata 17 software. For continuous outcomes, both the Mean Difference (MD) and the Standardized Mean Difference (SMD) were computed (12). The point estimates along with their corresponding 95% confidence intervals (95% CIs) were determined for each effect size calculated. Statistical significance was considered when the confidence interval did not cross the zero line, and the *p*-value was <0.05. In cases of statistical heterogeneity between studies (*I^2^* > 50% or *p* < 0.05), a random-effects model was applied to calculate the pooled outcome measures. Conversely, when there was no statistical heterogeneity (*I^2^* = 0%) or low-to-moderate heterogeneity (*I^2^* < 50%), a fixed-effects model was utilized for the analysis. In instances where substantial heterogeneity was observed among outcomes, a sensitivity analysis was performed to gauge the robustness of the findings. Additionally, the influence of each individual study on the overall results was examined by sequentially omitting each study to ascertain if any single study exerted a pronounced influence on the outcome.

## 3 Results

### 3.1 Selection and characteristics of studies

**Figure 1:**
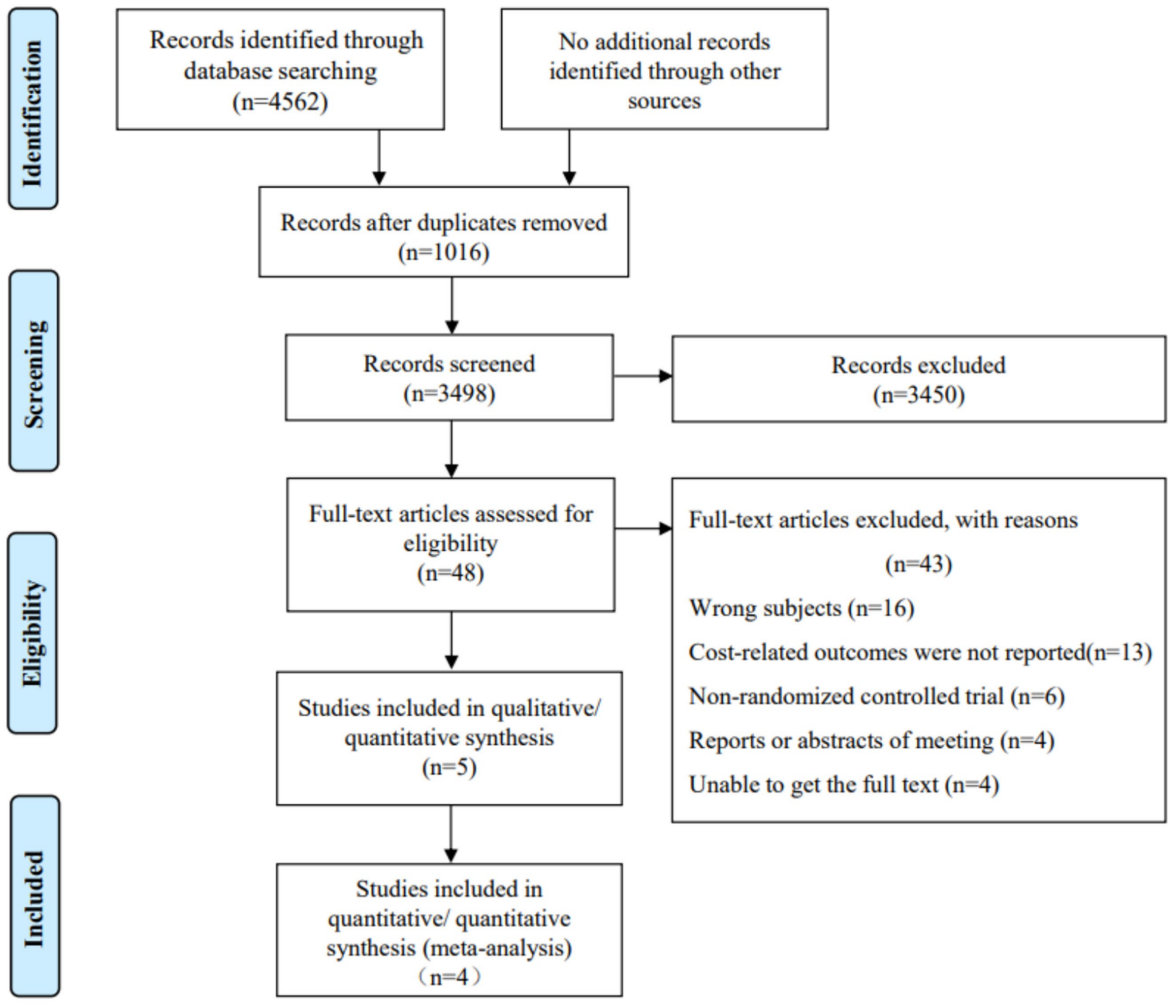
Flowchart of the study selection process.

The initial search yielded a total of 4,562 articles. After removing duplicates, 1,016 articles were excluded. Additionally, 3,498 articles were excluded based on title or abstract screening, and 48 were excluded after full-text review. Ultimately, 5 studies were included in the final analysis (Figure 1). All of the included studies were published between 2011 and 2023, and all were in English. The 5 studies involved 666 participants, with ages ranging from 60 to 75 years, and 51.2% of the participants were female (15–19). The patient population included individuals with various types of cancer and gynecological diseases, with some studies focusing on early-stage cancer or postoperative recovery. The interventions primarily involved robot-assisted minimally invasive surgeries, while the control groups consisted of traditional open surgeries or laparoscopic surgeries. Further details are provided in Table 1.

**Table 1:** Features of the included studies.

Study	Country	Patient	Sample size	Sex(*n*)Male/Female	Age (Mean ± SD; Range/years)	Outcomes
Dixon 2023 (15)	UK	Non-metastatic bladder cancer patients	iRARC (*n* = 157)	241/64	68.3 ± 8.1 year	Total cost, Operating room costs, Hospitalization expenses, Related benefits of QALY, Cost-Effectiveness/QALY
			ORC (*n* = 148)			
Lundin 2020 (16)	Sweden	Early-stage endometrial cancer patients	RH (*n* = 25)	0/49	68 (63–72)	Total cost, Operating room costs, Hospitalization expenses, Related benefits of QALY, Cost-Effectiveness/QALY
			AH (*n* = 24)		67 (59–75)	
Paraiso 2011 (17)	USA	Patients with vaginal prolapse after stage 2–4 hysterectomy	LS ( n = 38)	0/78	60 ± 11	Total cost, Operating room costs, Hospitalization expenses, Related benefits of QALY, Cost-Effectiveness/QALY
			RS ( n = 40)		61 ± 9	
Park 2012 (18)	Korea	Right-sided colon cancer patients	RAC ( n = 35)	30/40	62.8 ± 10.5	Total cost, Operating room costs, Hospitalization expenses, Related benefits of QALY, Cost-Effectiveness/QALY
			LAC ( n = 35)		66.5 ± 11.4	
Patel 2023 (19)	Canada	Early-stage non-small cell lung cancer patients	RPL-4 ( n = 81)	54/110	67 (60–74)	Total cost, Operating room costs, Hospitalization expenses, Related benefits of QALY, Cost-Effectiveness/QALY
			VATS ( n = 83)		68 (60–75)	

iRARC, Robot-Assisted Radical Cystectomy with Intracorporeal Urinary Diversion; ORC, Open Radical Cystectomy; RH, Robotic Hysterectomy; AH, Abdominal Hysterectomy; LSC, Laparoscopic Sacro colpopexy; RSC, Robotic Sacro colpopexy; RAC, Robot-Assisted Colectomy; LAC, Laparoscopically Assisted Colectomy; RPL-4, Robotic-Assisted Lobectomy; VATS, Video-Assisted Thoracic Surgery; QALY, Quality-Adjusted Life Year.

### 3.2 Quality assessment

**Figure 2:**
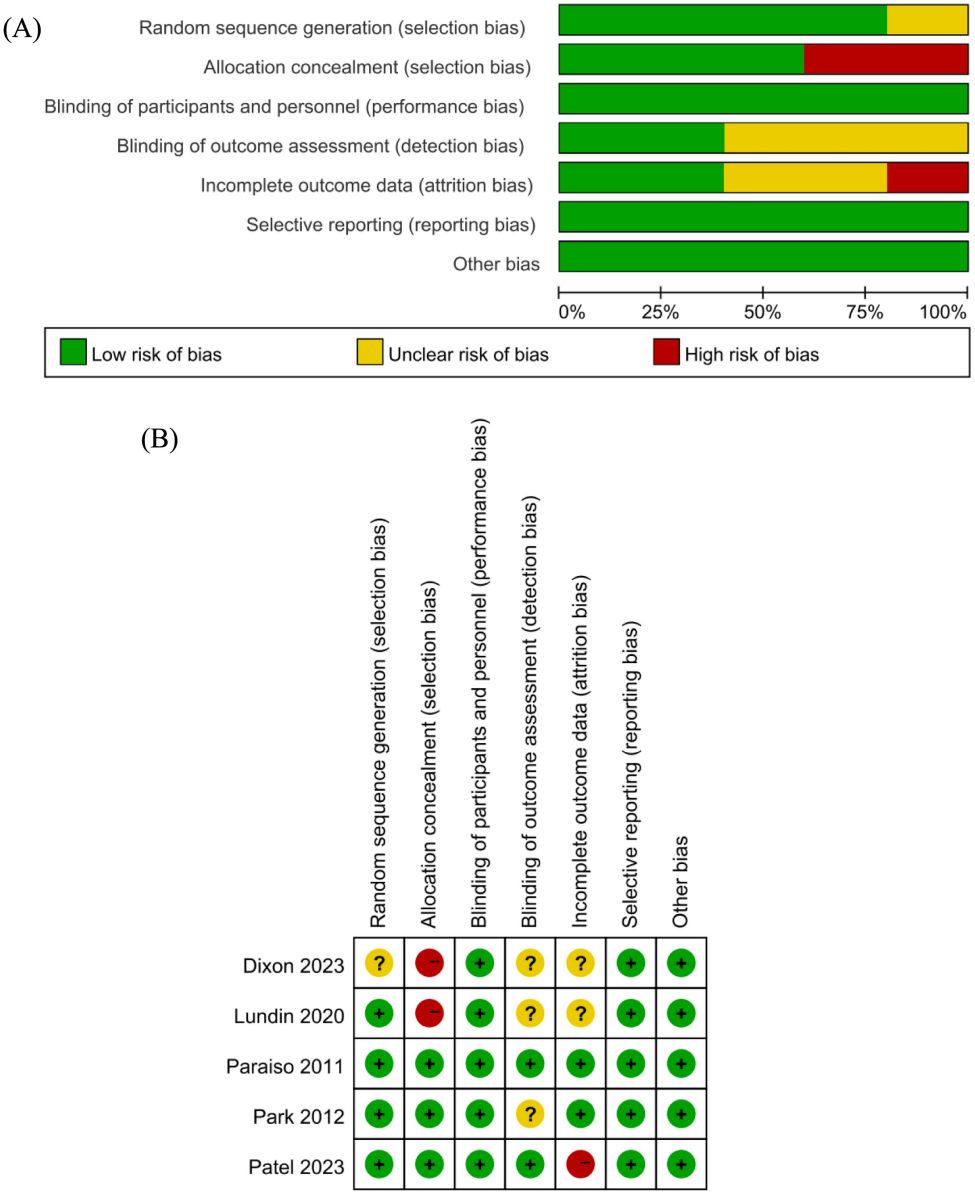
Risk of bias graph: **(A)** Risk of bias graph; **(B)** Risk of bias summary (“+” = Low risk of bias; “–” = High risk of bias; “?” = Unclear risk of bias).

Four studies documented their method of random sequence generation, with all of them employing random number tables to do so. Three studies detailed the allocation concealment method, while in the remaining 2 studies, participants were aware of their allocation. Three studies did not provide information on the completeness of outcome data, with 1 of these studies showing follow-up bias due to loss to follow-up. Two studies were judged to have no measurement bias, while the others did not clearly address this issue. All studies were rated as having no implementation bias, and all reported predefined outcomes without any other significant biases identified. In summary, the evaluation of the studies’ risk of bias yielded the following results: 1 study was deemed to have a low risk of bias, another study had an unclear risk, while 3 studies were categorized as having a high risk of bias. The detailed assessment of the risk of bias for the studies included in the analysis is illustrated in Figure 2.

### 3.3 Meta-analyses

#### 3.3.1 Total costs

**Figure 3:**
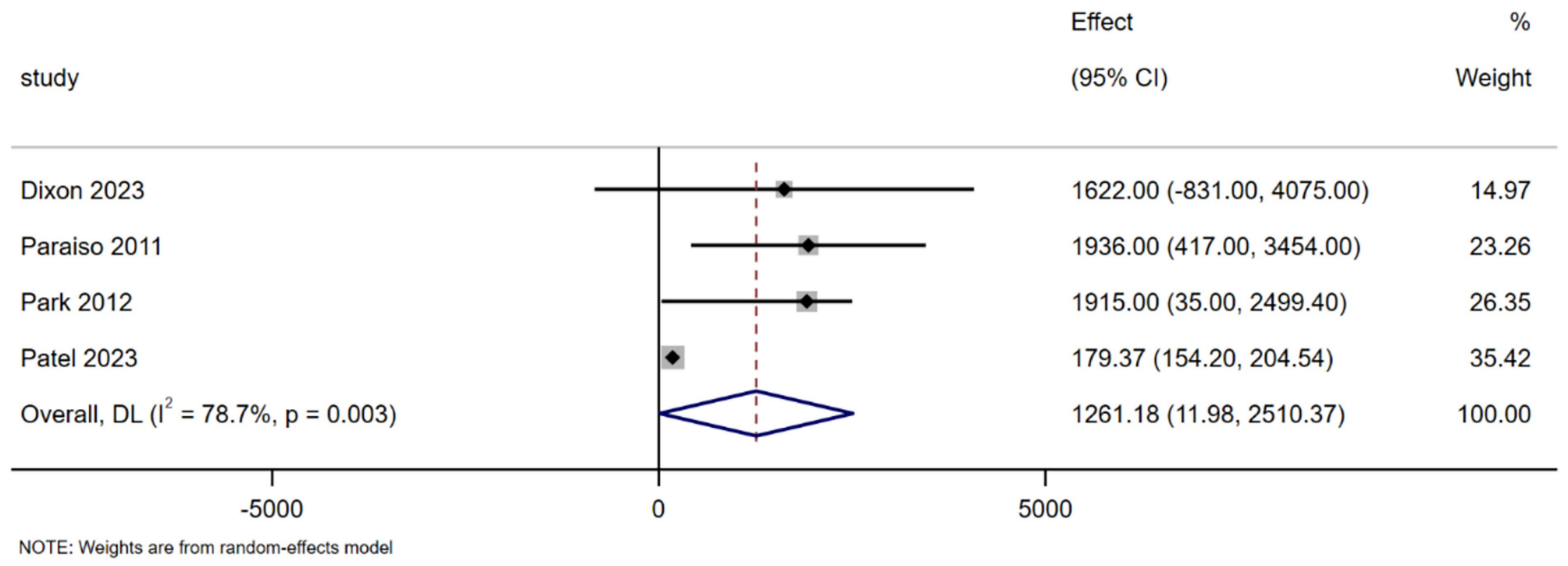
Forest plot of the total cost difference between the two interventions (USD).

Four studies, encompassing a total of 617 patients, were included in the MA assessing the overall costs of surgery (15–18). The combined results indicate that, during the treatment period, the total surgical costs for robot-assisted surgeries were higher compared to traditional open and laparoscopic surgeries in the intervention group (MD = 1316.38, 95% CI 10.68–2622.08; *p* = 0.048, *I*^2^ = 92.5%), with all differences being statistically significant. Significant heterogeneity was observed in the combined results (Figure 3). Furthermore, Report in a study, when the number of surgeries was 300, the cost difference per patient between robotic and traditional surgery was approximately $1,416.69. As the number of surgeries increased to 500, the cost difference decreased by 30%, with the difference per patient being $973.38 (19).

#### 3.3.2 Operating room costs

**Figure 4:**
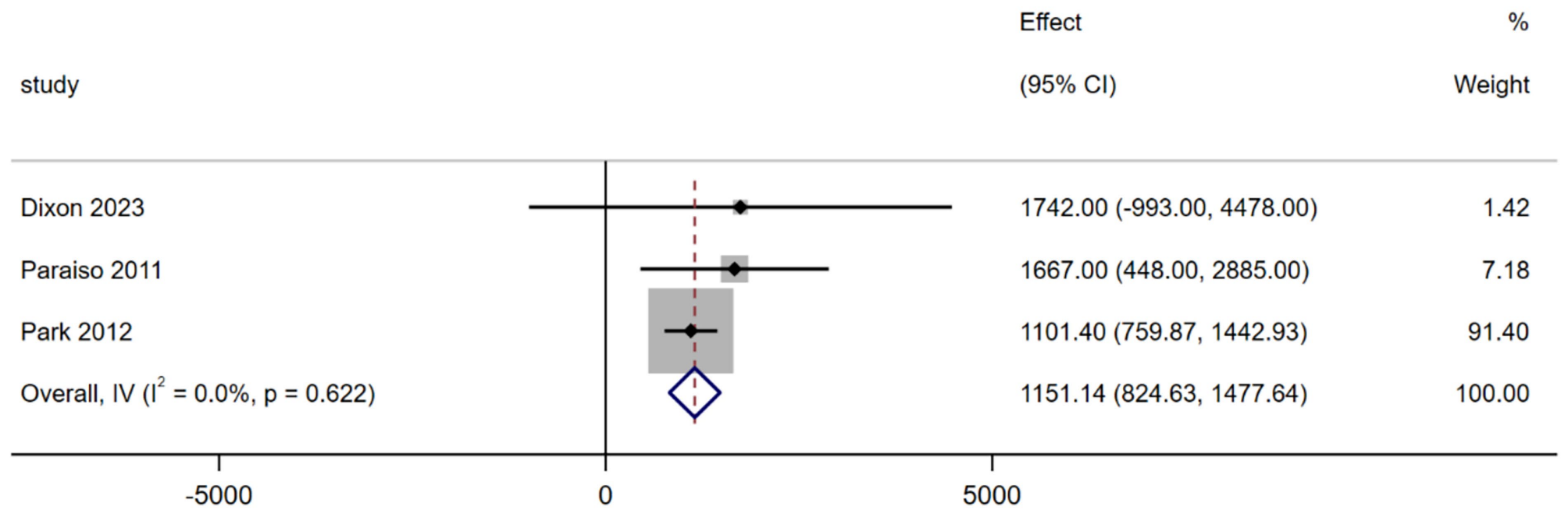
Forest plot of the operating room cost difference between the two interventions (USD).

Three studies analyzed the operating room equipment costs (15, 17, 18). The results of the MA, shown in Figure 4, indicate that the operating room costs for robotic surgery were considerably higher (MD = 1151.14, 95% CI 824.63–1477.64; *p* = 0.000, *I^2^* = 0.0%) compared to the traditional surgery group. All differences were statistically significant, and no heterogeneity was observed between the groups (Figure 4).

#### 3.3.3 Hospitalization costs

**Figure 5:**
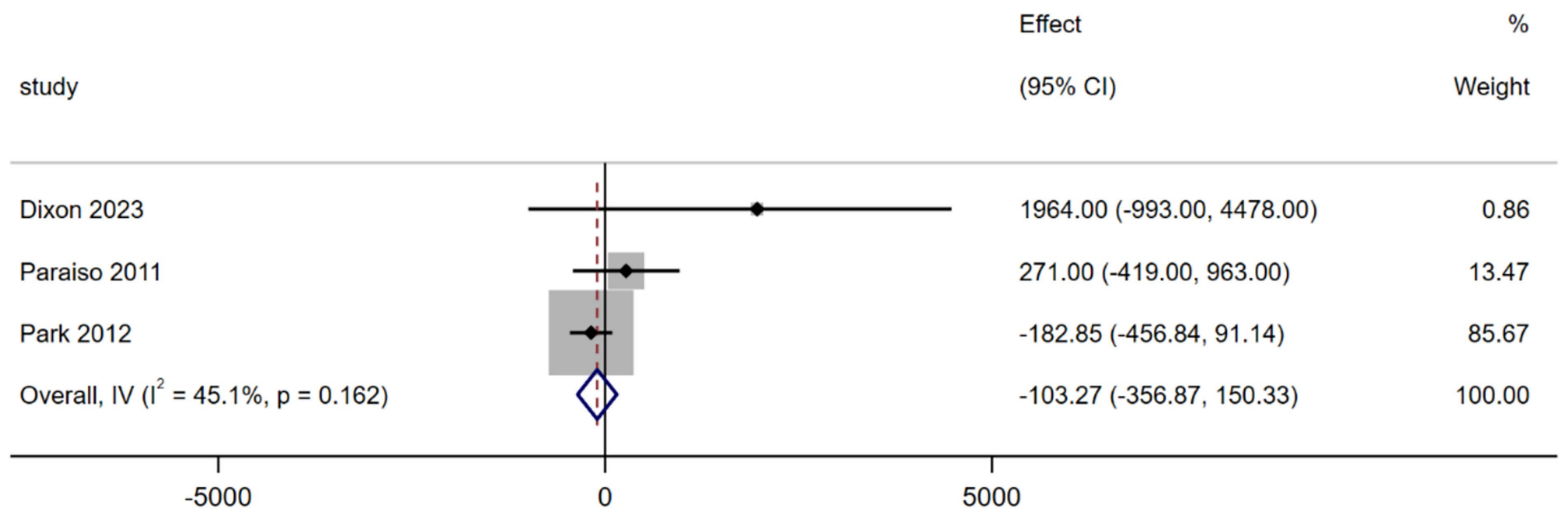
Forest plot of the hospitalization cost difference between the two interventions (USD).

Three studies reported hospitalization costs (15, 17, 18). The MA results indicated that, compared to the traditional surgery group, the robotic surgery group had lower hospitalization costs (MD = −103.27, 95% CI -356.87 to 150.33; *p* = 0.425, *I^2^* = 45.1%), however, the difference was not statistically significant, and moderate heterogeneity was observed across the studies (Figure 5).

#### 3.3.4 QALY-related gains

**Figure 6:**
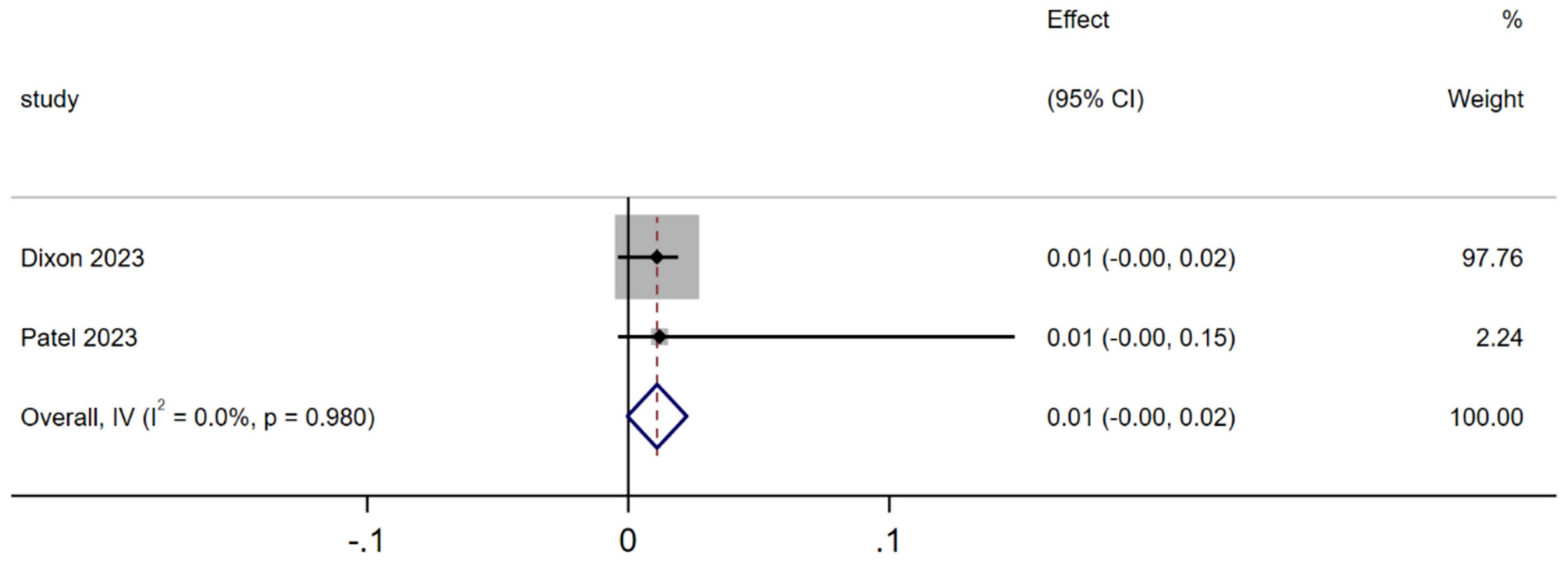
Forest plot of the QALY-related gain differences between the two interventions (QALY).

Two studies analyzed the QALY-related gains (15, 19). As shown in Figure 6, the MA results indicated that, compared to the traditional surgery group, the robotic surgery group had a statistically significant increase in QALY (MD = 0.01, 95% CI 0.00–0.02; *p* = 0.010, *I^2^* = 0.0%). The heterogeneity test revealed that there were no significant differences or variability among the studies included in the analysis.

#### 3.3.5 Incremental cost-effectiveness per QALY

Two studies reported the incremental cost-effectiveness per QALY (15, 19); however, due to inconsistencies in the reporting formats, a meta-analysis could not be performed. 1 study suggested that the cost-effectiveness of robotic surgery was £20,000 ($28,860) for each additional quality-adjusted life year (QALY) gained, with a likelihood of 61.9% that it is cost-effective. For patients aged 70 years and above, the probability that the intervention would be cost-effective at the threshold of $28,860 per QALY was 82.2%. A separate study found that the additional cost for each QALY gained with RPL-4 was $14,925.62 (with a 95% confidence interval ranging from $6843.69 to $23,007.56) at the 12-month, a figure that is significantly lower than the commonly accepted cost-effectiveness threshold of $50,000.

### 3.4 Assessment of sensitivity and publication bias

**Figure 7:**
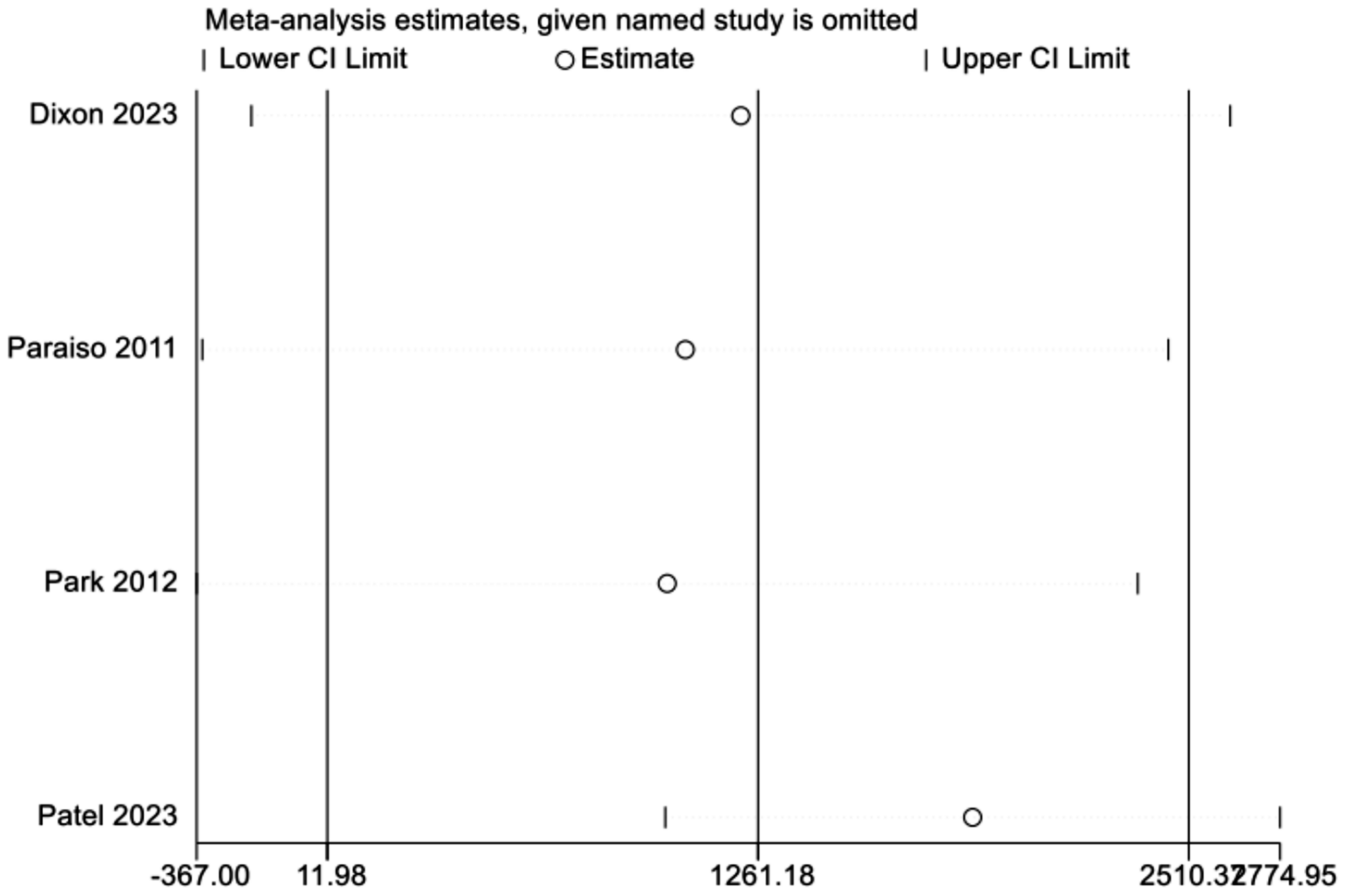
Sensitivity analysis plot.

In examining the total cost outcome, a sensitivity analysis was conducted by systematically removing each study one by one. The findings revealed that 1 study was a significant source of heterogeneity (19). Upon the exclusion of this study, the level of heterogeneity was markedly diminished (MD = 1903.77, 95% CI 1371.38–2436.18; *p* = 0.000, *I^2^* = 0.0%) (Figure 7). Given that fewer than 10 studies were included in the meta-analysis, and in accordance with Cochrane recommendations, we did not perform Egger’s test or construct a funnel plot. The small number of studies limits the ability to reliably assess publication bias using visual or statistical methods, and this should be considered when interpreting the results.

## 4 Discussion

This study conducted a SR/MA based on data from RCTs to evaluate the cost-effectiveness of surgical robots in healthcare management for older patients. In total, 5 studies involving 666 patients were included, aiming to assess the cost performance of surgical robots in the treatment of older individuals.

The results showed that the overall treatment costs were higher for robotic surgery compared to traditional surgery. In sensitivity analysis, excluding 1 specific study further increased the observed cost differences. Upon further investigation, the limited cost difference in that study was attributed to the minimal variation in robotic equipment costs (19). This finding suggests that the costs of surgical robots vary significantly depending on their functions and intended applications. To improve cost-effectiveness in clinical practice, surgical robots with relatively lower equipment costs should be prioritized for wider adoption, balancing technological benefits with economic feasibility. Further subgroup analysis of total costs revealed that the primary contributor to cost differences was the significantly higher operating room expenses, while differences in hospitalization costs were relatively minor. Specifically, the high initial investment required for robotic surgery was mainly reflected in equipment procurement and maintenance costs. Higgins et al. (8) noted that while robotic surgery offers clinical advantages, the overall treatment costs often exceed those of traditional surgery due to higher consumable expenses. This issue is particularly pronounced in developing countries, where the high costs of robotic surgery limit its widespread application. Compared to traditional laparoscopic surgery, robotic surgery requires significantly greater investments in equipment procurement and technical training. This cost structure may make it challenging to maximize cost-effectiveness in certain scenarios2 (7). However, as technology advances and clinical experience accumulates, the costs associated with robotic surgery are gradually decreasing (20). Studies have shown that the long-term use of robotic surgical techniques can help reduce operating time and hospitalization duration, thereby lowering overall healthcare costs (21). For complex diseases in particular, robotic surgery can significantly improve treatment outcomes, reduce postoperative complications, and subsequently lower readmission rates and long-term treatment costs (22). For example, the application of robotic-assisted surgery in older cancer patients has been shown to enhance surgical precision and reduce postoperative complications, resulting in lower long-term treatment expenses (23).

SR/MA often consider patient quality of life as a key metric when evaluating the cost-effectiveness of robotic surgery. Robotic surgery has demonstrated a notable ability to improve quality of life by increasing treatment success rates and reducing side effects. This is particularly important for older patients, who often face longer recovery periods due to their unique physiological conditions. Interventions that improve quality of life tend to gain greater support (24, 25). In this study, the analysis of QALY gains showed a statistically significant for robotic surgery compared to traditional surgery. This indicates that robotic-assisted surgery has potential benefits in enhancing quality of life and extending healthy lifespan. Specifically, robotic surgery improves functional recovery by minimizing complications through precise operations, thereby increasing QALY. In our study, the included trials primarily involved older adult populations (mean ages ranging from 64.7 to 71.3 years), many of whom had multiple comorbidities such as cardiovascular disease, diabetes, or impaired mobility. These baseline characteristics likely amplify the impact of postoperative outcomes—such as complication rates, recovery time, and hospital length of stay—on long-term quality of life. The observed QALY gains reported in the included studies were largely attributable to improvements in key postoperative outcomes. These included reduced complication rates, shorter length of stay, faster return to baseline function, and improved symptom control—particularly with respect to pain, mobility, and self-care. These domains were measured using validated HRQoL instruments such as EQ-5D and SF-6D, and informed utility values used in the economic evaluations. Such findings suggest that the cost-effectiveness of robotic surgery may be closely linked to its capacity to mitigate functional decline and accelerate recovery, particularly in older adults. This finding aligns with existing literature, which suggests that robotic technology significantly reduces recovery time and improves patient quality of life (26, 27). However, despite its statistical significance, the observed QALY gain from robotic surgery was relatively small, at only 0.01 years. This suggests that while robotic surgery’s potential to improve quality of life should not be overlooked, the clinical relevance of this improvement requires longer follow-up periods and larger datasets for validation. Future research should further explore the long-term QALY gains of robotic surgery in older individuals, especially in patients with complex conditions, to better understand its impact on quality of life.

Moreover, 2 studies reported the incremental cost-effectiveness per QALY (15, 19); however, due to inconsistencies in data presentation, the results could not be pooled. Nevertheless, individual analyses of these studies provided valuable insights. 1 study reported an incremental cost of £20,000 (approximately $28,860) per QALY gained for the robotic surgery group, with a cost-effectiveness probability of 61.9% (15). This finding suggests that robotic surgery offers a certain degree of cost-effectiveness. Additionally, this study specifically analyzed patients aged 70 years and older, reporting that the incremental cost-effectiveness ratio (ICER) of iRARC was $28,860 per QALY, with a probability of 82.2% in this subgroup. These data suggest that robotic surgery may provide greater cost-effectiveness for older patients, particularly for those in higher age groups, aligning with findings from other studies (28). Another study reported an incremental cost per QALY of $14,925.62 (95% CI: $6,843.69–$23,007.56), which is well below the commonly accepted cost-effectiveness threshold of $50,000 (19). This result further supports the economic feasibility of robotic surgery, particularly in the context of long-term treatment. It is important to note that the $50,000 per QALY threshold, commonly cited in U. S.-based economic evaluations, is not a globally accepted benchmark. Cost-effectiveness thresholds vary widely across countries, often depending on gross domestic product (GDP) per capita, healthcare budget constraints, and national health policy. For instance, the World Health Organization has suggested using one to three times the GDP per capita as a threshold in low- and middle-income countries, while some high-income countries, such as the UK, apply a lower threshold (e.g., £20,000–£30,000 per QALY used by NICE). These differences should be considered when interpreting the cost-effectiveness of robotic interventions in different health system contexts.

In countries with a high proportion of older individuals, such as Japan and Germany, policy environments have been relatively supportive of integrating robotic surgery into routine practice. For example, Japan’s National Health Insurance began reimbursing selected robotic procedures in 2018 under strict credentialing and institutional requirements, aiming to ensure safe and cost-effective implementation in the face of demographic pressures. Modeling studies have suggested that maintaining high annual surgical volumes is crucial for achieving cost-efficiency in such settings (29, 30). In contrast, low- and middle-income countries (LMICs) face significant challenges, including high initial investment costs, limited infrastructure, and a lack of trained personnel. In these settings, implementation strategies may need to prioritize cost-effective robotic applications (e.g., high-volume cancer surgeries), establish regional centers of excellence, and explore public–private partnerships to offset investment burdens (31). Additionally, policy frameworks should incorporate long-term planning to assess not only direct clinical outcomes but also broader system-level effects, such as workforce implications, training demands, and equity in access to robotic care.

Regarding methodological quality, the majority of included studies showed notable concerns in several key domains of bias. While most studies clearly described random sequence generation and were therefore considered at low risk of selection bias in this domain, allocation concealment was inadequately reported in two studies, potentially compromising the internal validity of group assignment. Blinding of participants and personnel was generally not feasible due to the nature of surgical interventions; however, the lack of assessor blinding in several studies may have introduced detection bias, particularly for outcomes such as hospitalization costs and QALY gains, which are susceptible to subjective influence. Incomplete outcome data also posed a potential risk in three studies that failed to address loss to follow-up adequately or lacked intention-to-treat analysis. These high-risk studies contributed to the observed heterogeneity in the total cost meta-analysis. These findings underscore the importance of rigorous trial design and transparent reporting in economic evaluations of robotic interventions.

Based on the current evidence, robotic surgery demonstrates a certain degree of economic viability in terms of incremental cost-effectiveness, especially in improving QALY for older patients. While some studies have highlighted the high initial investment required for robotic surgery, the long-term cost-effectiveness is increasingly evident with advancements in technology and the accumulation of clinical experience. Future research should focus on analyzing the specific applications of different types of surgical robots in older individuals, particularly for patients with chronic diseases or multiple comorbidities. In parallel, it is also important to explore the cost-effectiveness of other robotic technologies such as rehabilitation robots, socially assistive robots, and those used in non-surgical therapeutic contexts (which were not included in this analysis)—including physical rehabilitation, cognitive-behavioral therapy, speech therapy, and occupational support—which may provide substantial long-term value by improving functional recovery, reducing reliance on human caregiving, and promoting independent living. However, during the screening process of this review, we observed that many studies on these non-surgical robots did not meet the inclusion criteria, largely due to non-randomized designs, lack of economic outcomes (e.g., QALY or cost-effectiveness), or early-stage technological development. This highlights a notable gap in the literature. Future research should therefore prioritize high-quality trials and comprehensive economic evaluations in these areas to inform the broader integration of robotic surgery into diverse healthcare settings and better support aging populations. Furthermore, the evaluation of the cost-effectiveness of robotic surgery should not be limited to a simple numerical comparison but should also incorporate multiple factors such as patients’ specific health conditions, treatment needs, and acceptable costs. Future studies should adopt a multidimensional evaluation framework that considers treatment outcomes, costs, quality of life, and other relevant factors to provide more comprehensive decision-making support.

### 4.1 Limitations

Indeed, while this study offers important initial insights into the cost-effectiveness of robotic surgery for older patients, it is pertinent to recognize its limitations: First, the limited number of included studies and inconsistencies in data reporting formats may have affected the integration and analysis of results. Second, varying degrees of bias were identified among the 5 included studies, particularly in areas such as randomization, allocation concealment, data completeness, and measurement bias, which may impact the reliability of the findings. Third, most of the included studies only reported short-term follow-up outcomes, typically within 12 to 24 months post-intervention. There is a lack of long-term cost-effectiveness data (i.e., more than 5 years), which limits the ability to evaluate sustained economic value, such as reductions in rehospitalization, late complications, or long-term quality of life improvements. Future studies should incorporate longer follow-up periods to better inform healthcare investment decisions. Finally, the conclusions of this study are based on literature published up to November 2024. As robotic technologies continue to evolve—particularly with the integration of artificial intelligence, machine learning, and next-generation automation—new economic evidence is likely to emerge. The current review may not fully reflect the cost-effectiveness profiles of these emerging systems. Therefore, regular updates of this evidence synthesis will be essential to ensure its ongoing relevance and accuracy in informing policy and clinical decisions.

## 5 Conclusion

The application of robotic surgery in older individuals’ health management holds significant potential to improve treatment outcomes, reduce postoperative complications, and shorten recovery times. However, the high costs associated with robotic technology remain a major barrier to its widespread adoption. With continued advancements in technology and the accumulation of clinical experience, the costs of robotic therapy are expected to gradually decline, offering patients better treatment outcomes and improved quality of life. To support the broader integration of robotic therapy into healthcare systems, policymakers should consider establishing clear health insurance coverage standards, developing standardized clinical protocols, and ensuring equitable access to cost-effective robotic solutions. In addition, multinational comparative studies are needed to generate context-specific evidence and guide cross-country policy alignment. Future research should continue to explore the clinical and economic value of robotic technologies in older individuals and inform long-term investment strategies.

## Data Availability

The datasets used and/or analyzed during the current study available from the corresponding author on reasonable request.
