# Supplementary material for: Cost-effectiveness analysis of robotic surgery in healthcare for older individuals: a systematic review based on randomized controlled trials
Source: Front Public Health. 2025 Aug 12;13:1614654. doi: 10.3389/fpubh.2025.1614654 (PMC12381784; doi:10.3389/fpubh.2025.1614654)
Supplement: Supplementary file 1 [file Table_1.docx]

**Appendix A:**

**Table 1 Search Strategy**

| **PubMed** |
| --- |
| #1 aged[Mesh]  #2 elderly[Title/Abstract] OR "older adults"[Title/Abstract] OR "older patients"[Title/Abstract] OR "seniors"[Title/Abstract]  #3 #1 OR #2  #4 paro[Title/Abstract] OR robot*[Title/Abstract] OR "social interactive robot*"[Title/Abstract] OR "assistive robot*"[Title/Abstract] OR "companion robot*"[Title/Abstract] OR "social commitment robot*"[Title/Abstract] OR "robot* therapy"[Title/Abstract] OR "therapeutic robot*"[Title/Abstract] OR "robot interaction"[Title/Abstract] OR "personal robot*"[Title/Abstract] OR "therapeutic seal robot*"[Title/Abstract]  #5 randomized controlled trial[Title/Abstract] OR controlled clinical trial[Title/Abstract] OR clinical trials[Title/Abstract] OR randomly trial[Title/Abstract] OR randomized[Title/Abstract] OR placebo[Title/Abstract]  #3 AND #4 AND #5 |
| **Cochrane** |
| #1 MeSH descriptor: [aged]  #2 elderly OR "older adults" OR "older patients" OR seniors  #3 paro or robot* or "social interactive robot*" or "seal robot" or "assistive robot*" or "personal assistive robot*" or "social interactive robot*" or "assistive robot*" or "companion robot*" or "social commitment robot*" or "robot* therapy" or "therapeutic robot*" or "robot interaction" or "personal robot*" or "therapeutic seal robot*"  #4 randomized controlled trial OR controlled clinical trial OR clinical trials OR randomly trial OR randomized OR placebo  #5 #1 OR #2  #6 #5 AND #3 AND #4 |
| **WOS** |
| #1 TS= ("aged" OR "elderly" OR "older adults" OR "older patients" OR "seniors")  #2 TS= (paro or robot* or "social interactive robot*" or "seal robot" or "assistive robot*" or "personal assistive robot*" or "social interactive robot*" or "assistive robot*" or "companion robot*" or "social commitment robot*" or "robot* therapy" or "therapeutic robot*" or "robot interaction" or "personal robot*" or "therapeutic seal robot*")  #3 TS= (randomized controlled trial OR controlled clinical trial OR clinical trials OR randomly trial OR randomized OR placebo)  #4 #1 AND #2 AND #3 |
| **Embase** |
| #1 'aged'/exp OR 'elderly'/exp OR 'older adults'/exp OR 'older patients'/exp OR 'seniors'/exp  #2 'paro'/exp OR 'robot*'/exp OR 'social interactive robot*'/exp OR 'seal robot'/exp OR 'assistive robot*'/exp OR 'personal assistive robot*'/exp OR 'social interactive robot*'/exp OR 'assistive robot*'/exp OR 'companion robot*'/exp OR 'social commitment robot*'/exp OR 'robot* therapy'/exp OR 'therapeutic robot*'/exp OR 'robot interaction'/exp OR 'personal robot*'/exp OR 'therapeutic seal robot*'/exp  #3 'randomized controlled trial'/exp OR 'controlled clinical trial'/exp OR 'randomly trial'/exp OR 'randomized'/exp OR 'placebo'/exp  #4 #1 AND #2 AND #3 |
| **CNKI** |
| #1老人 + 老年人 + 年长患者 + 老年  #2机器人 + 手术机器人 + 辅助机器人  #3'随机对照试验' + '干预对照' + '临床实验'  #4 #1 AND #2 AND #3 |
| **CBM** |
| #1 "老人" [常用字段: 智能] OR "老年人" [常用字段: 智能] OR "年长患者" [常用字段: 智能] OR "老年" [常用字段: 智能]  #2 "机器人" [常用字段: 智能] OR "手术机器人" [常用字段: 智能] OR "辅助机器人"  #3 "随机对照试验" [常用字段: 智能] OR "干预对照" [常用字段: 智能] OR "临床实验" [常用字段: 智能]  #4 AND #2 AND #3 |
| **Wangfang** |
| #1老人or老年人or 烟雾 or年长患者or老年  #2机器人or手术机器人or辅助机器人  #3"随机对照试验" or "干预对照" or "临床实验"  #4 AND #2 AND #3 |
| **VIP** |
| #1老人 + 老年人 + 年长患者 + 老年  #2机器人 + 手术机器人 + 辅助机器人  #3'随机对照试验' + '干预对照' + '临床实验'  #4 #1 AND #2 AND #3 |

**Table 2 Quality assessment results of the included studies**

| Study | Random sequence generation | Allocation concealment | Blinding of participants and personnel | Blinding of outcome assessment | Incomplete outcome data | Selective outcome reporting? | Other bias |
| --- | --- | --- | --- | --- | --- | --- | --- |
| Dixon 2023 | Unclear | High | Low | Unclear | Unclear | Low | Low |
| Lundin 2020 | Low | High | Low | Unclear | Unclear | Low | Low |
| Paraiso 2011 | Low | Low | Low | Low | Low | Low | Low |
| Park 2012 | Low | Low | Low | Unclear | Low | Low | Low |
| Patel 2023 | Low | Low | Low | Low | High | Low | Low |
